# Non contrast enhanced volumetric histology of blood clots through high resolution propagation-based X-ray microtomography

**DOI:** 10.1038/s41598-022-06623-8

**Published:** 2022-02-17

**Authors:** Somayeh Saghamanesh, Daniela Dumitriu LaGrange, Philippe Reymond, Isabel Wanke, Karl-Olof Lövblad, Antonia Neels, Robert Zboray

**Affiliations:** 1 Center for X-Ray Analytics, Swiss Federal Laboratories for Materials Science and Technology (Empa), 8600 Dübendorf, Switzerland; 2 Neuroradiology Division, Diagnostic Department, University of Geneva, Geneva, Switzerland; 3 Neuroradiology Division, Klinik Hirslanden, Zurich, Switzerland; 4 Faculty of Medicine, University of Geneva, Geneva, Switzerland

**Keywords:** X-ray tomography, Computed tomography, Electron microscopy, Nanocrystallography, Stroke

## Abstract

We have demonstrated the capability of laboratory propagation-based microtomography (miroCT) in non-destructive 3D virtual histopathology of human blood clots without any contrast agent. The volumetric information are valuable to understand the mechanical properties of clots which are crucial in selecting the most efficient mechanical thrombectomy method for clot extraction. Different clot types retrieved by mechanical thrombectomy from patient victims of acute ischemic stroke were evaluated through propagation-based microCT. The results were correlated with high-resolution scanning electron microscopy (SEM) images, confirming detected cellular and fibrillary structures. Calcifications appeared as glassy opacity areas with relatively intense signal on microCT images, also proved by energy-dispersive spectroscopy and X-ray diffraction. Hyperintense regions on the microCT corresponded to individual or compact aggregates of red blood cells, whereas fibrin dominated volumes appeared at consistently moderate to low normalized microCT values. Red blood cell shapes and sizes are consistent with the SEM observations. Together with other potential parameters, 3D porosity distribution and volume fraction of structures can be easily measured by microCT data. Further development of automated post-processing techniques for X-ray propagation-based micro/nanoCT, also based on machine learning algorithms, can enable high throughput analysis of blood clot composition and their 3D histological features on large sample cohorts.

## Introduction

The examination of ex vivo histology of clots extracted by mechanical thrombectomy (MTB) from patient victims of acute ischemic stroke (AIS) is necessary in several instances, for example when making correlative links with the pre-interventional clot migration^1^, with the treatment outcome^2^, or when identifying clinical imaging features predictive for the treatment outcome^3^. The mechanical properties of the thrombus including the stiffness play an important role in the efficacy of MTB and this was proved to have a strong relationship with the clot composition^4^.

Conventionally, the examination of the clot histology is performed using optical microscopy on stained 2D slices cut from paraffin-embedded samples. Most commonly, the clot histology is classified based on the red blood cell (RBC)-to-fibrin ratio either dichotomously, as red (RBC rich) and white (fibrin rich)^5^, or in three ordinal categories (red, mixed, and white)^6^. However, when using conventional 2D histopathology, the clot categorization is subjected to inherent uncertainties because it relies on the examination of 2D slices, which cannot account for the overall volumetric composition due to intra-clot heterogeneity. Quantifying the clot composition, when using conventional histopathology through serial 2D depiction, is a difficult and time consuming task. Recently, optical clearing methods were reported as such that 3D renderings of stained clots can be obtained with optical microscopy^7^. However, technical limitations impede this method from becoming effective in reconstructing the entire clot sample, e.g. the inability to image beyond 1 mm depth while keeping the required resolution for the structures of the clot (RBCs, fibrin); and the confinement to a limited field of view. Inherent inaccuracies in clot classification can affect the output of statistical analysis studies, where the volumetric clot composition is an important parameter^3^. Therefore, developing volumetric analysis methods becomes necessary. Such methods can enable an accurate assessment of the clot composition or the ratio of its main components and provide a new tool for clot composition classification.

X-ray phase-contrast imaging (XPCI) techniques have demonstrated significant improvement in the image contrast specifically for soft tissues suffering from the low sensitivity for conventional X-ray attenuation imaging^8^. Among those techniques, propagation-based imaging (PBI) is a simple and efficient non-contact X-ray imaging method that is sensitive to phase-shifts of X-ray wave fronts upon penetration and refraction through the matter when propagating to a sufficient distance to the detector^9^. This technique enables higher contrast micro and nanotomography (microCT/nanoCT) and structural analysis down to a sub-micrometer scale^10^.

In this study, we demonstrate the capability of X-ray propagation-based (PB) microCT on a few samples from three ordinal clot categories to capture blood clot composition and structural features. We will address the validity, potential, and limitations of this technique. Our purpose is to investigate if PB microCT can serve as a high-throughput, noninvasive, digital 3D histopathology technique for the evaluation of blood clot composition.

## Results

Here we augment and validate our X-ray PB microCT with other imaging and analytical techniques providing complementary advantages. X-ray diffraction (XRD) enables analysis at atomic scales, whereas scanning electron microscopy (SEM) can typically work down to the single digit nanometer range. We give explicit examples in this section as well as in the Supplementary Information (SI), how cellular (RBCs) and fibrillary structures clearly resolved by high-resolution SEM images are associated and correlated with structures revealed by X-ray PB microCT.

### Case 1

**Figure 1:**
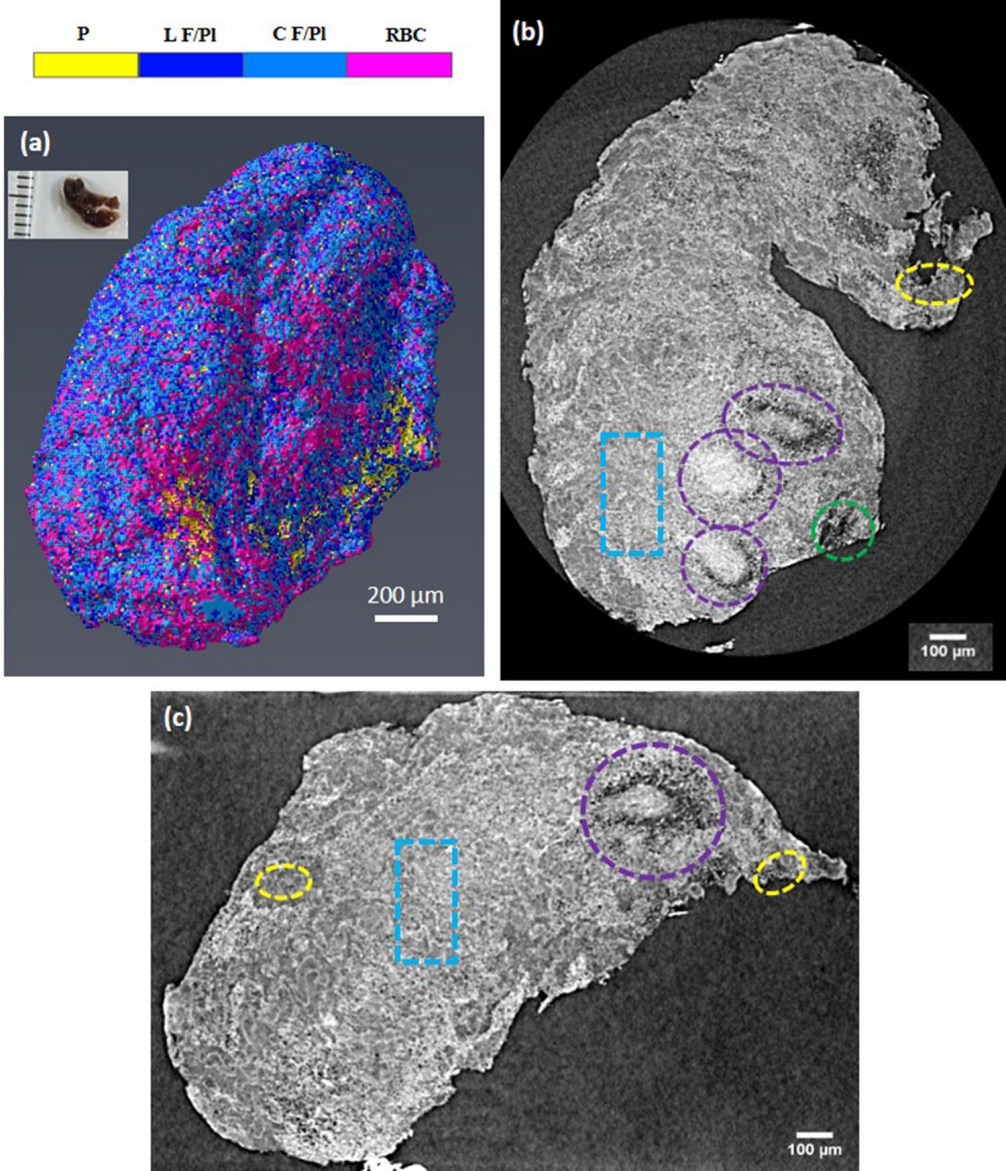
Reconstructed MicroCT slices for sample 1. (**a**) Snapshot of the 3D rendering of sample 1. Inset in (**a**): a photograph of the clot fixed in formalin. (**b**,**c**) selected microCT slices along two orthogonal directions to illustrate typical regions with different texture and structures. Colorbar: colors attributed to different structures based on the gray level distribution: RBC, red blood cells; L F/Pl, loose and porous fibrin and platelets; C F/Pl, compact fibrin/platelets; P, porosity. In Avizo (2019.4): https://www.thermofisher.com/amira-avizo.

The representative depictions of microCT data for clot sample 1 are shown in Fig. 1, which held a red appearance. Figure 1a presents the 3D rendering of sample 1, while Fig. 1b,c shows tomography reconstructed slices selected from two orthogonal views within the sample, each at different depths from the same stack of images. Massive hyperintense regions are distinguished within microCT slices, surrounded by low intensity structures (purple dashed circles in Fig. 1b,c).

The gray levels in the low intensity regions are comparable with those of the air surrounding the sample, which suggest pore volumes in different sizes inside the clot. As observed from the data acquired at different depths within the sample, the hyperintense volume regions represent large inclusions, hundreds of micrometers in average size throughout the sample body with tens of micrometers, in average, porous boundaries. The main body of the sample consists of a medium intensity marbled by a hyperintense network (blue dashed rectangles), as exemplified in Fig. 1b,c. Infrequent low-intensity regions with no or scarce hyperintense content are also observed (yellow and green dashed circles in Fig. 1b,c).

**Figure 2:**
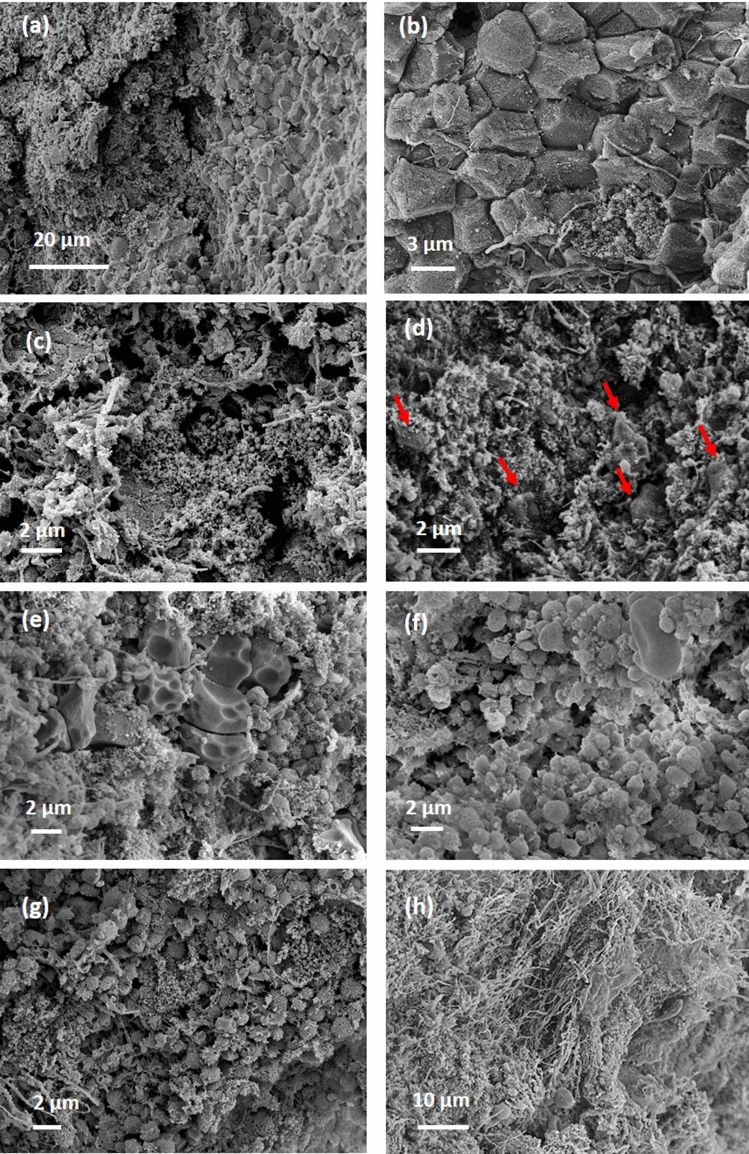
Scanning electron micrographs, representative for the structure and composition of sample 1. (**a**) agglomerates of red blood cells with porous boundaries, corresponding to areas marked with purple contour in Fig. 1. (**b**,**c**) higher magnification of red blood cell aggregates and porous boundaries. (**d**,**e**) typical composition for the main body, marked with blue contour in Fig. 1. (**f**,**g**) typical composition for regions marked with yellow contour in Fig. 1. (**h**) fibrin strands found in the region marked with green contour in Fig. 1. Red arrows point towards red blood cells. Generated by ZeissSmartSEM software (version 6.06).

In order to assign various intensity regions from microCT slices to specific histological features, we sectioned the sample and examined it with SEM (Fig. 2). Consistently, the hyperintense inclusions with porous boundaries were found to be large compact aggregates of polyhedrally shaped red blood cells, while the porous boundaries were found to be fibrillar strands intercalated with globular debris, most likely protein content. An exemplification of these hyperintense masses is presented in Fig. 2a,b,c. The main body of the sample (blue rectangles in Fig. 1b,c) consists of a compact aggregate of similar fibrillar structures with debris and variable platelet content in which red blood cells are firmly encased, either single or clustered together, as confirmed in Fig. 2d,e. We attribute the regions with low intensity in microCT (e.g. yellow circles in Fig. 1b,c) to loose, porous aggregates of fibrins and platelets with infrequent biconcave red blood cells, as observed in Fig. 2f,g. We assign the region marked with green circle in Fig. 1b to long fibrin strands, probably an extension of the fibrin clot’s outer surface in the clot main body (see Fig. 2h). According to above assignment of different intensity-range regions to observed structures, we can estimate the volumetric content of them in the clot, calculated as a percentage of the whole clot volume. These data together with other measured parameters for all clots are presented in Table 1.

**Table 1:** MicroCT analysis of three measured clot samples: volume fraction (Vol) and calibrated mean intensity value (MI) for each segmented structure.

Segmented structures	Sample 1		Sample 2		Sample 3	
	Vol (%)	MI	Vol (%)	MI	Vol (%)	MI
RBC	9.74	1925	8.09	1936	17.46	5662
C F/Pl	42.62	1404	46.54	1393	53.64^a^	1773
L F/Pl	37.91	975	34.48	1013	–	–
P	9.73	507	10.9	511	4.98	544
Ca	–	–	–	–	23.91	3074

*RBC* red blood cells, *C F/Pl* compact fibrins/platelets/proteins, *L F/Pl* loose, porous fibrin/platelets, *P* porosity, *Ca* calcifications.

^a^In this sample, due to the spread of unexpected intense signal, a distinction between compact and loose fibrins were not possible. Also, areas of nano-calcifications and fibrin/platelets and RBCc could be interlaced.

**Figure 3:**
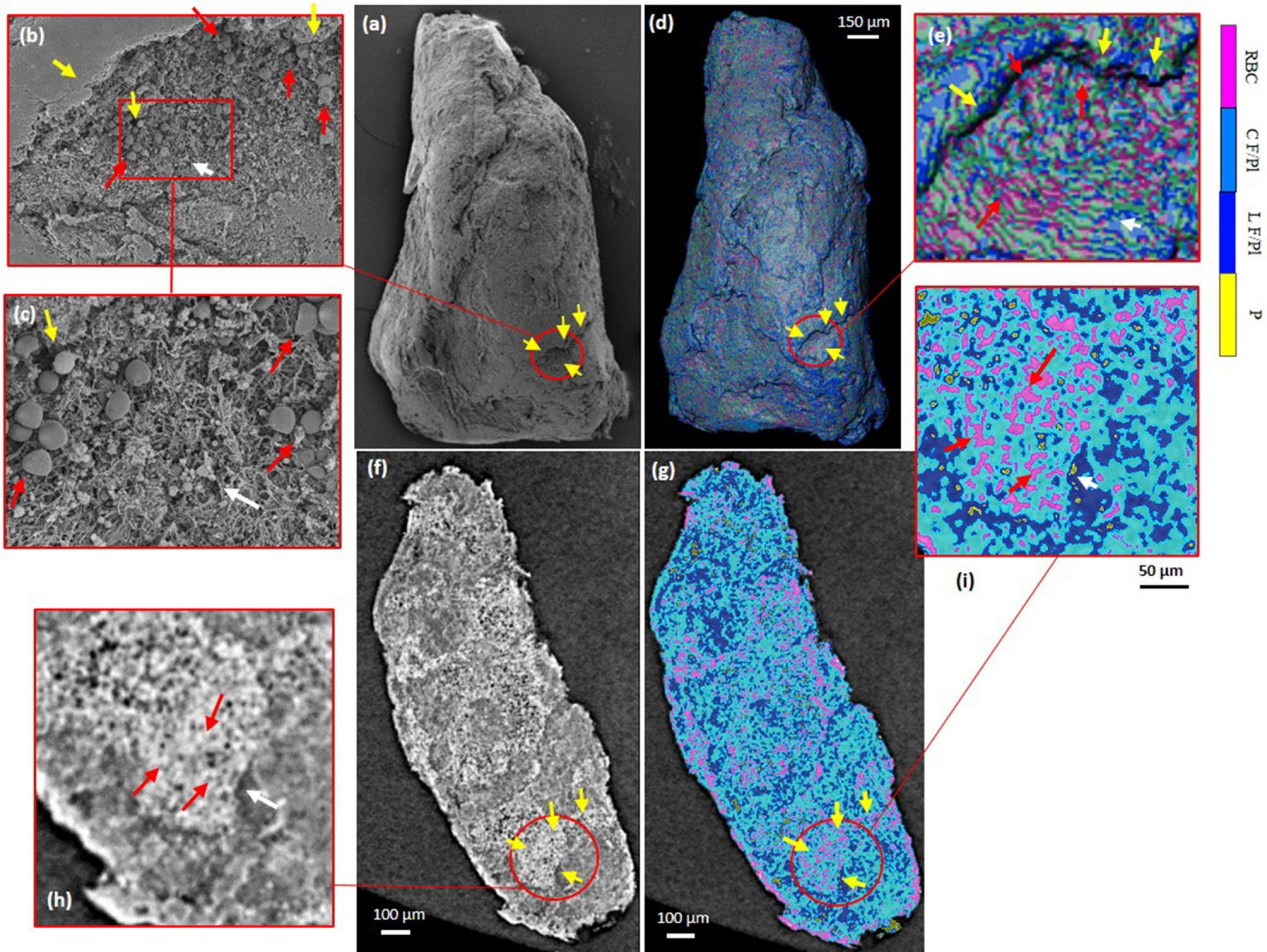
Co-registeration of 2D and 3D microCT data with SEM images for sample 1. (**a**) SEM image of the clot. (**b**) zoomed ROI of the red circle in (**a**). (**c**) zoomed ROI from red rectangle in (**b**). (**d**) 3D rendering snapshot of the segmented microCT volume. (**e**) zoomed ROI of red circle in (**d**). (**f**) corresponding 2D reconstructed slice close to the surface including the red circled ROI in (**d**). (**g**) segmented slice from (**f**). (**h**) zoomed ROI of red circle in (**f**). (**i**) zoomed ROI of red circle in (**g**). Yellow arrows show some fiducial structures in the co-registration. Red arrows indicate RBCs. White arrows indicate fibrin/platelet structures close to the pores. Colorbar: RBC, red blood cells; C F/Pl, compact fibrin/platelets; L F/Pl, loose and porous fibrin/platelets; P: porosity. In Avizo (2019.4): https://www.thermofisher.com/amira-avizo.

An example of an approximate co-registration between microCT data and SEM images is presented in Fig. 3 for sample 1. In this figure, a common region of interest (ROI) has been determined through fiducial structures (yellow arrows) on the SEM as well as the 3D-rendered segmented volume of the clot 1 (Fig. 3a,d). This ROI has been examined closer in higher magnified images of SEM and microCT volume (Fig. 3b,c,e). In addition, the microCT slices were reconstructed again on a parallel plane clipped on the surface of the clot so that it included the ROI (Fig. 3f) and then it was segmented (Fig. 3g). A higher magnification of the ROI are shown in Fig. 3h,i. By tracking marked fiducial structures on SEM images and comparison with similar ROI on microCT images, RBC and fibrin/platelet distributions can correspondingly found and confirmed.

MicroCT voxel size for this sample was 1.23 µm, although it has a spatial resolution less than SEM but it is principally expected to discern individual RBC with an average size of 4–7 µm or RBC masses, as indicated by red arrows in Fig. 3e,i and confirmed by the same ROI on SEM (Fig. 3b,c). See also Supplementary Fig. S1.

### Case 2

**Figure 4:**
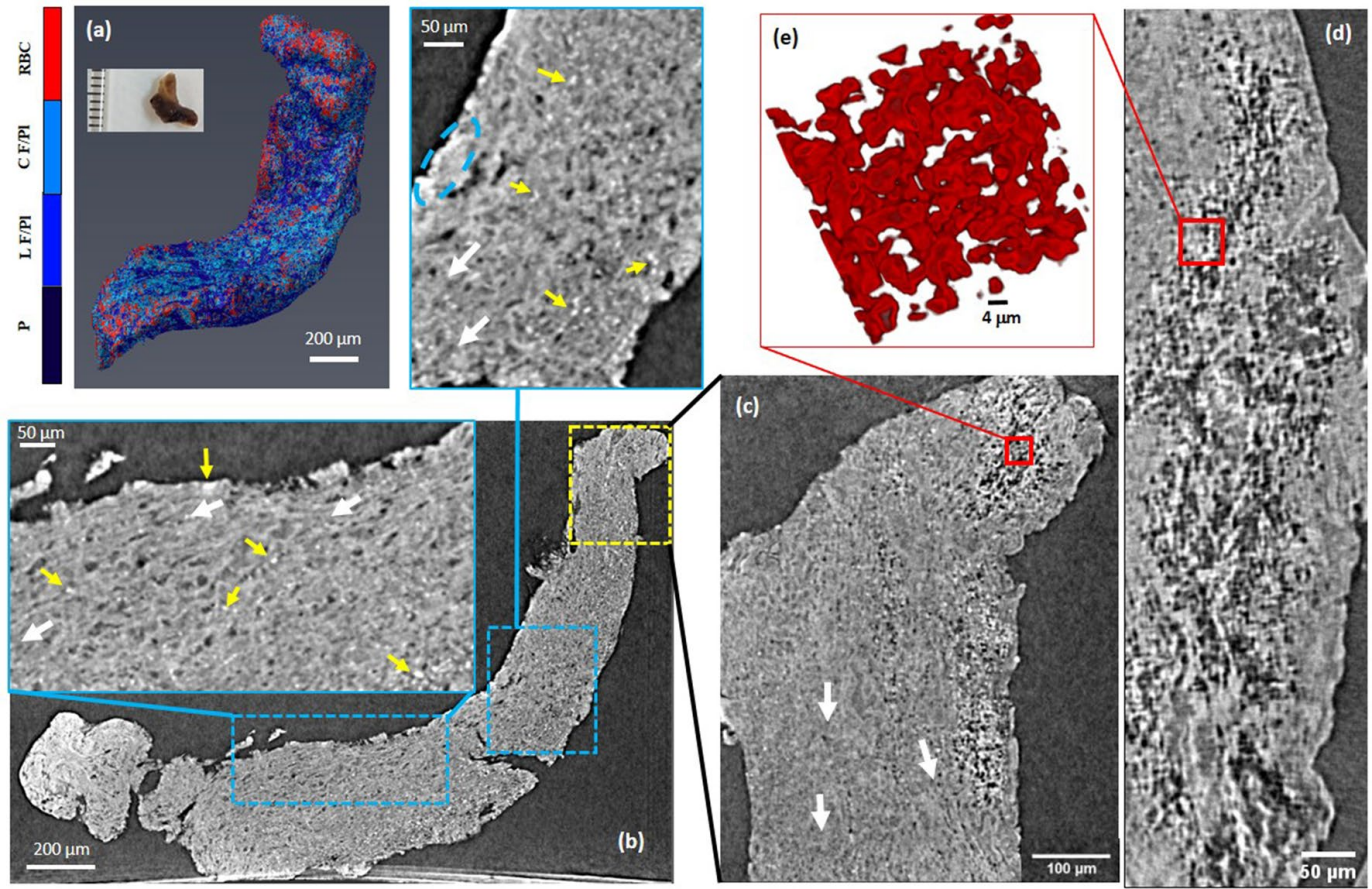
Reconstructed microCT slices for sample 2. (**a**) Snapshot of the 3D rendering of sample 2. Colorbar annotations are similar to Figs. 1 and 3. Inset in (**a**): a photograph of the clot fixed in formalin. (**b**) MicroCT slice with zoomed ROIs. Blue lines point to the enlarged views of each contour. White arrows indicate observed structural orientations. Yellow arrows point to hyperintense masses (RBCs). (**c**,**d**) Orthogonal slices from local nanoCT of the yellow dashed rectangle area in (**b**). (**e**) A 3D rendering of individual and clustered RBCs with their typical biconcave shapes from a very small ROI volume (red squares in (**c**) and (**d**)). It is a region with relatively high porosity, nonetheless, RBCs are often attached and form clusters. In Avizo (2019.4): https://www.thermofisher.com/amira-avizo.

The reconstructed microCT data for sample 2 with a mixed appearance is presented in Fig. 4. A 3D rendering of the clot volume is depicted in Fig. 4a, and slices selected in orthogonal directions are shown in Fig. 4b,c. The microCT slices at the scale of the full clot appear relatively uniform in sample 2, however, zooming in to smaller ROIs reveal quite some structures. For example, regions of hyperintensity are observed throughout the slices, as shown in the inset of Fig. 4b (dashed blue contours), as well as on the outer surface of the sample. These hyperintense regions are more clear on the local tomography slice from the yellow contour (Fig. 4b) in Fig. 4c,d. It is worth to notice that a periodicity in gray levels is observed throughout the sample, suggesting the organization of the clot structure along preferential directions (white arrows in the insets of Fig. 4b,c).

**Figure 5:**
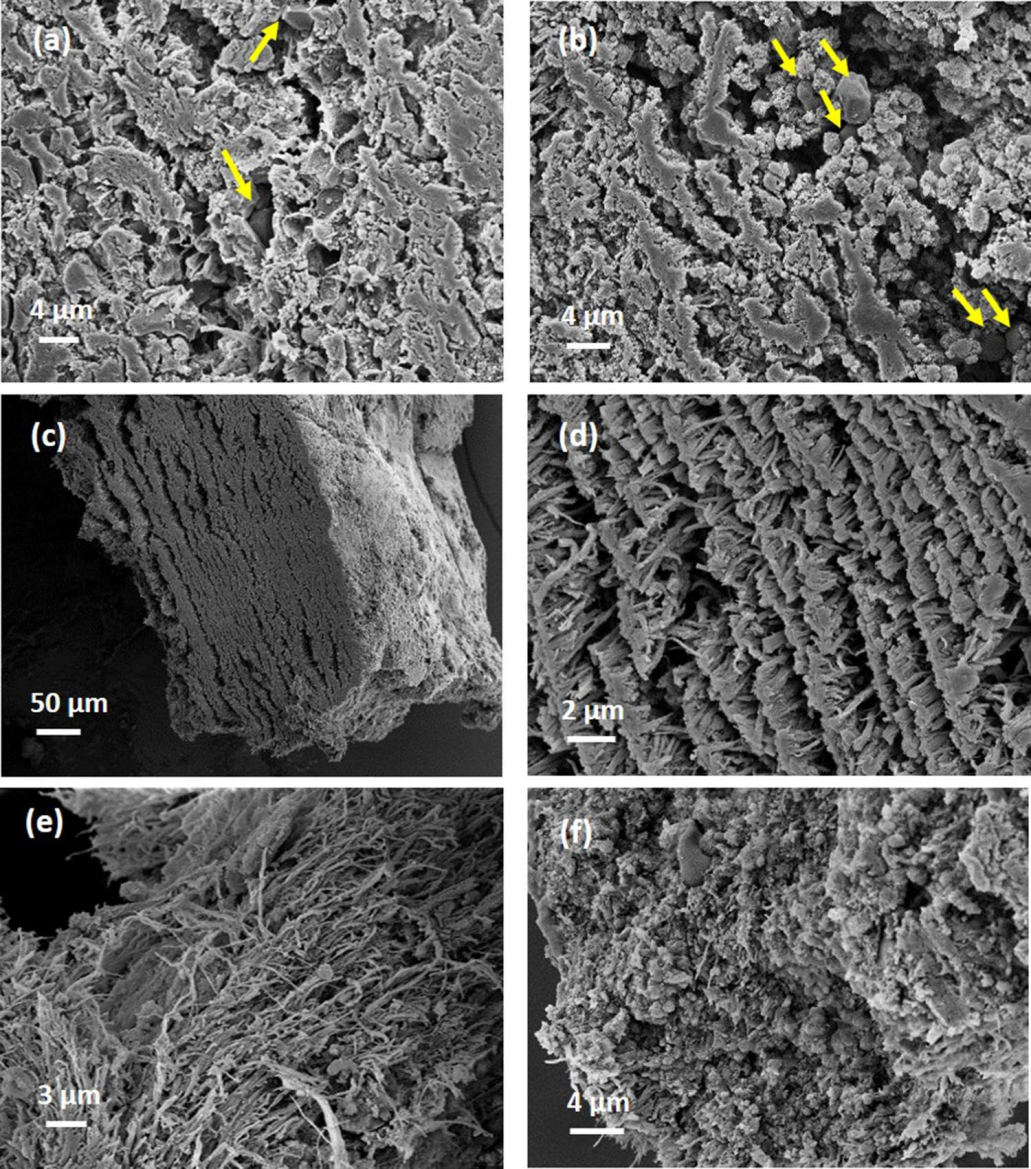
Scanning electron micrographs for sample 2. (**a**,**b**) representative structures for the region marked with yellow contour in Fig. 4. (**c**,**d**) layered fibrin, at two different magnifications. (**e**,**f**) fibrin strands and platelets corresponding to the region marked with blue dashed oval in Fig. 4. Yellow arrows point towards red blood cells. Generated by ZeissSmartSEM software (version 6.06).

The detailed examination of sample 2 by SEM images in Fig. 5a,b shows a compact stratification of platelets with scattered and tightly packed red blood cells on one end of the sample (corresponding to Fig. 4c,d). Moreover, macrophages are also observed, although infrequently, in Fig. 5a,b. Indeed, as exemplified in Fig. 5c,d, the microCT regions with periodicity of gray levels correspond to a layered structure of fibrin strands. Deviation from the periodicity occurs in regions in which the layered structure is interrupted, either by fibrin bundles not participating in the layered organization or by agglomerates of platelets and protein material (see Fig. 5e,f). Having the same strategy for the segmentation as the case sample 1, we assigned the hyperintense regions in the microCT images to RBCs as well as the compact and loose fibrin-platelets regions correspondingly. Measured quantities are reported in Table 1.

### Case 3

**Figure 6:**
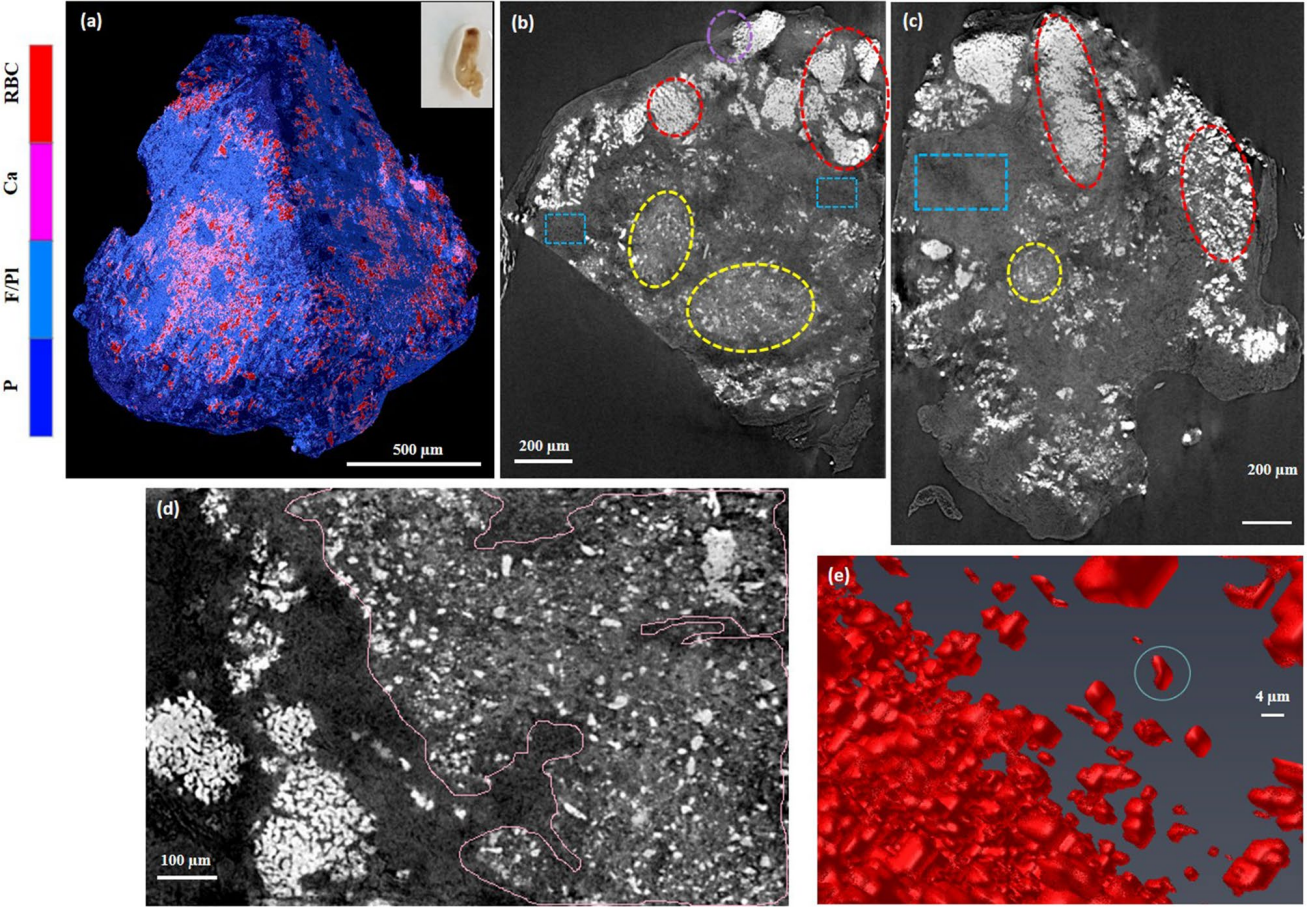
MicroCT results for sample 3. (**a**) Snapshot of 3D rendering of the clot. Inset in (**a**): a photograph of the clot fixed in formalin. (**b**,**c**) Reconstructed microCT slices along two orthogonal directions. (**d**) Local nanoCT of a ROI on the sample. Pink contour contains areas with the most visible glassy background (nano-calcifications). (**e**) 3D rendering of the hyperintense masses (RBCs). Colorbar: Ca denotes the glassy opacity regions (calcifications); F/Pl is fibrin/platelet-rich areas regardless of the density distribution. In Avizo (2019.4): https://www.thermofisher.com/amira-avizo.

The clot in sample 3 had a white appearance. Figure 6a shows a snapshot from the 3D rendering of the clot volume. A selected number of reconstructed microCT slices of sample 3 are shown in Fig. 6b,c. The slices reveal large agglomerates of compact hyperintense areas (volume regions—red dashed contours) despite the white appearance of the clot. The sample body exhibits diffuse regions of lower, variable intensity (blue dashed contours in Fig. 6b,c). In addition, a glassy opacity with unresolvable structure are clearly visible throughout the clot volume mostly spread around the hyperintense sparse masses (yellow dashed contours in Fig. 6b,c and pink contour in Fig. 6d), which was not observed in two previous samples. These regions form an evenly distributed area and have a gray level higher than the sample body but lower than the compact hyperintense area. The Detailed numerical results of this clot are reported in Table 1.

**Figure 7:**
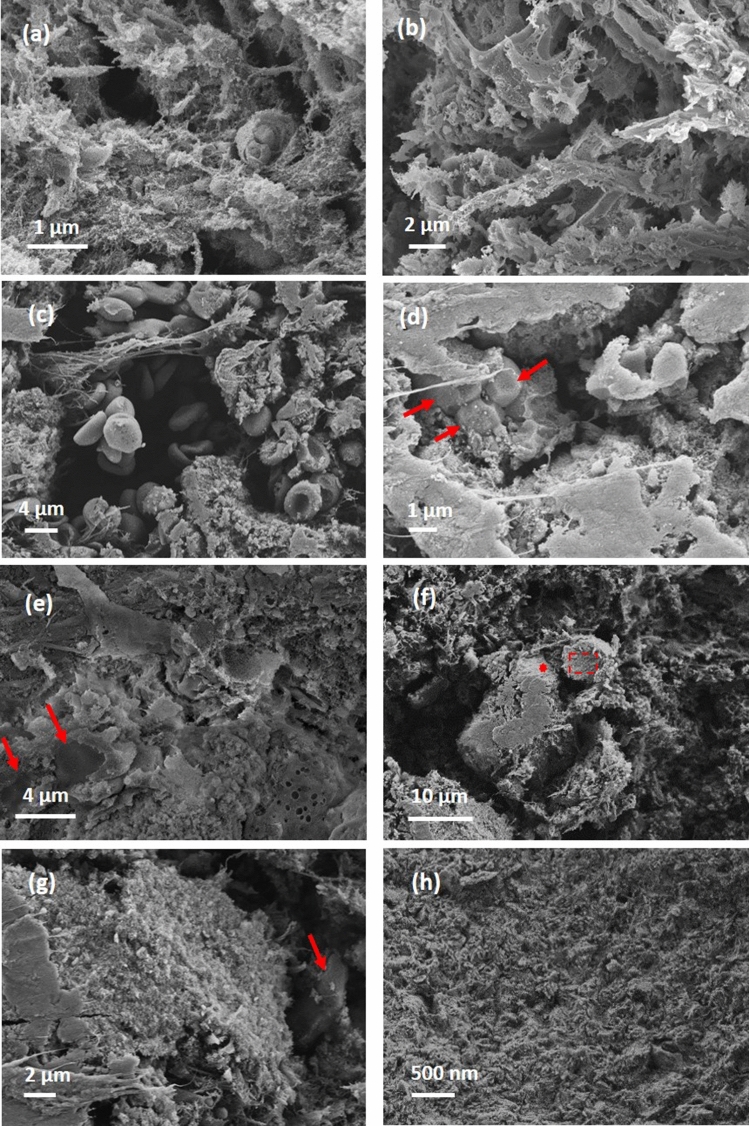
Scanning electron micrographs for sample 3. (**a**,**b**) Representative structures for blue contour in Fig. 6. (**c**) purple contour in Fig. 6. (**d**) red contour in Fig. 6. (**e**,**f**) yellow contour in Fig. 6. (**g**,**h**) higher magnification view of typical aggregates with polycrystalline appearance. (**g**) and (**h**) correspond to red star and red dashed rectangle in (**f**), respectively. Red arrows point towards red blood cells. Generated by ZeissSmartSEM software (version 6.06).

The examination of the areas with the lowest intensity (blue dashed contours in Fig. 6b,c) by SEM reveals a porous sponge-like structures (see Fig. 7a,b). The pore size ranges from micron to nanometer level. We detected no red blood cells content in the areas of lowest intensity. However, in pits close to the clot outer surface (purple dashed contour in Fig. 6b), we observed biconcave red blood cells scattered among strings of fibrin, as seen in Fig. 7c. In the hyperintense areas (red dashed contour in Fig. 6), the SEM images unveil a compact structure with encased red blood cells, as observed in Fig. 7d. NanoCT tomography indicates for the hyperintense regions compact aggregates of shapes and sizes consistent with the SEM observations (Fig. 6d,e). There is a relatively decreased compactness in the areas with intermediate intensity (yellow dashed contour in Fig. 6), as also seen in Fig. 7e,f.

**Figure 8:**
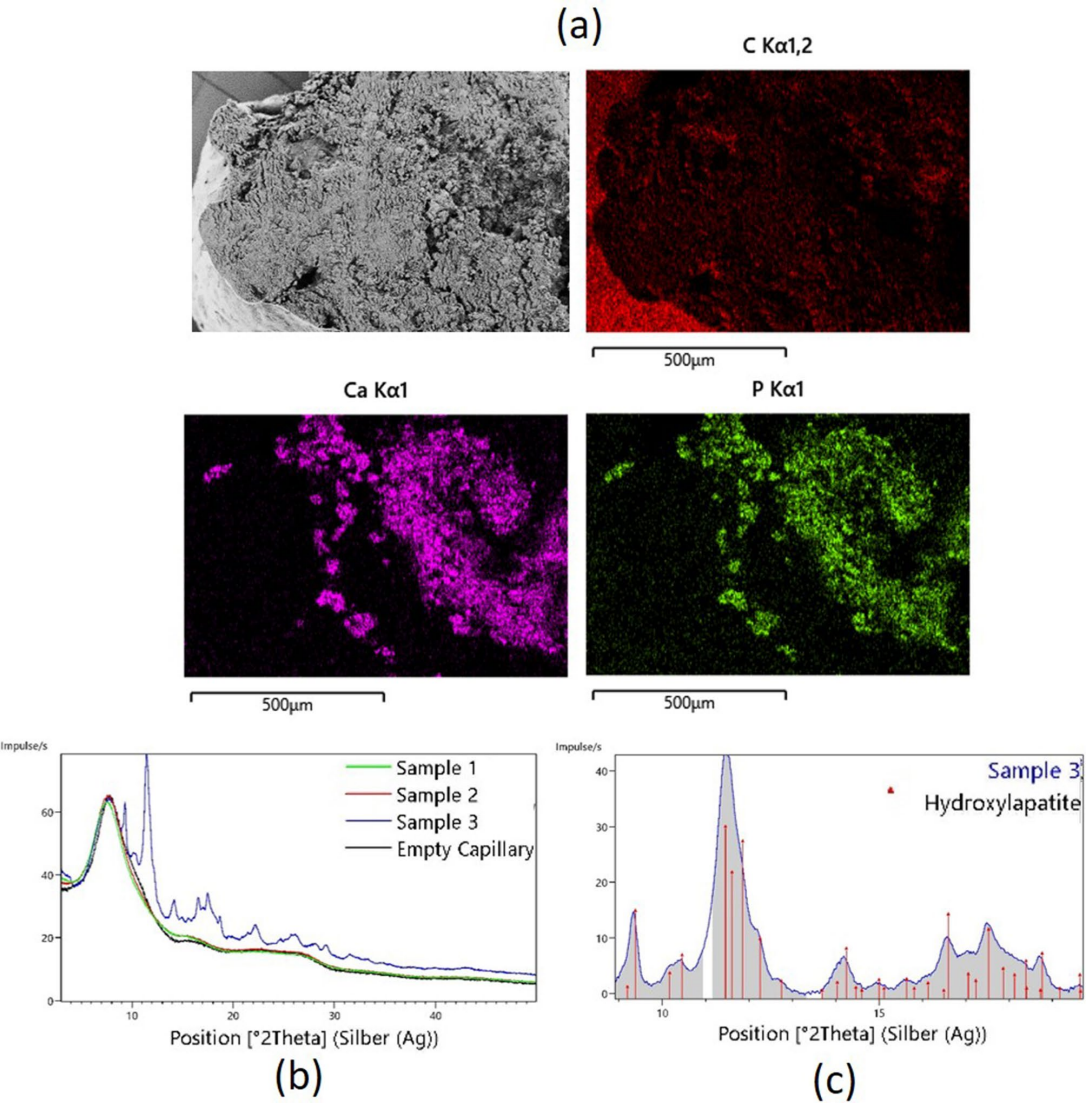
(**a**) SEM EDS of a cross section of sample 3 with relevant EDS mapping corresponding to C, Ca, and P Kα emission; recorded by AZtec 5.0 software (OXFORD Instruments) (**b**) XRD patterns for the three samples^15,16^. For sample 3, the XRD pattern is presented in blue. For sample 1, 2 and the holder: green, red, and black. (**c**) Indexing of the XRD pattern obtained for sample 3, with hydroxyapatite Ca10(OH)2(PO4)6 (PDF: 96-901-1093, COD database^21^).

However, along with the spongiform material and the red blood cells, there are forms of a polycrystalline appearance, which can be confirmed in Fig. 7g,h. Since the sample 3 appeared as white despite of a high portion of detected RBC regions and also unknown intense attenuation signals were found, we used energy dispersive spectroscopy (EDS) and XRD to check whether sample 3 contains calcifications. According to the results of SEM EDS performed on a cross section of this clot sample, regions rich in calcium (Ca) were found which coincided with regions rich in phosphorus (P) (see Fig. 8). No indication of iron was observed which is not surprising, given the iron concentration in the rarely found red blood cells (about 4 × 10^5^ ions per red blood cell^11^), the dispersed state of iron sites and the small peak-to-background ratio expected.

Because of the porosity and heterogeneous structural organization of the sample, these results hold a qualitative value, by giving an indication of the sample volume region enriched in calcium while a quantitative interpretation is not feasible by EDS. However, the XRD analysis confirms the presence of hydroxyapatite in sample 3 (blue diffraction curve in Fig. 8b). The indexing for the detected hydroxyapatite (Fig. 8c) correlates with the EDS indication for Ca and P. The average crystallite size calculated from XRD is 17 ± 4 nm. The XRD results didn't suggest the presence of Ca-containing minerals in sample 1 and 2 based on the absence of crystalline phases such as hydroxyapatite (green and red diffraction curves in Fig. 8b), as agreed with microCT analysis as well. We believe that the presence of calcifications, interlaced with fibrin-rich and hyperintense regions and even porosity in sample 3, contributed to the increased local signal of those regions for this sample (Table 1).

**Figure 9:**
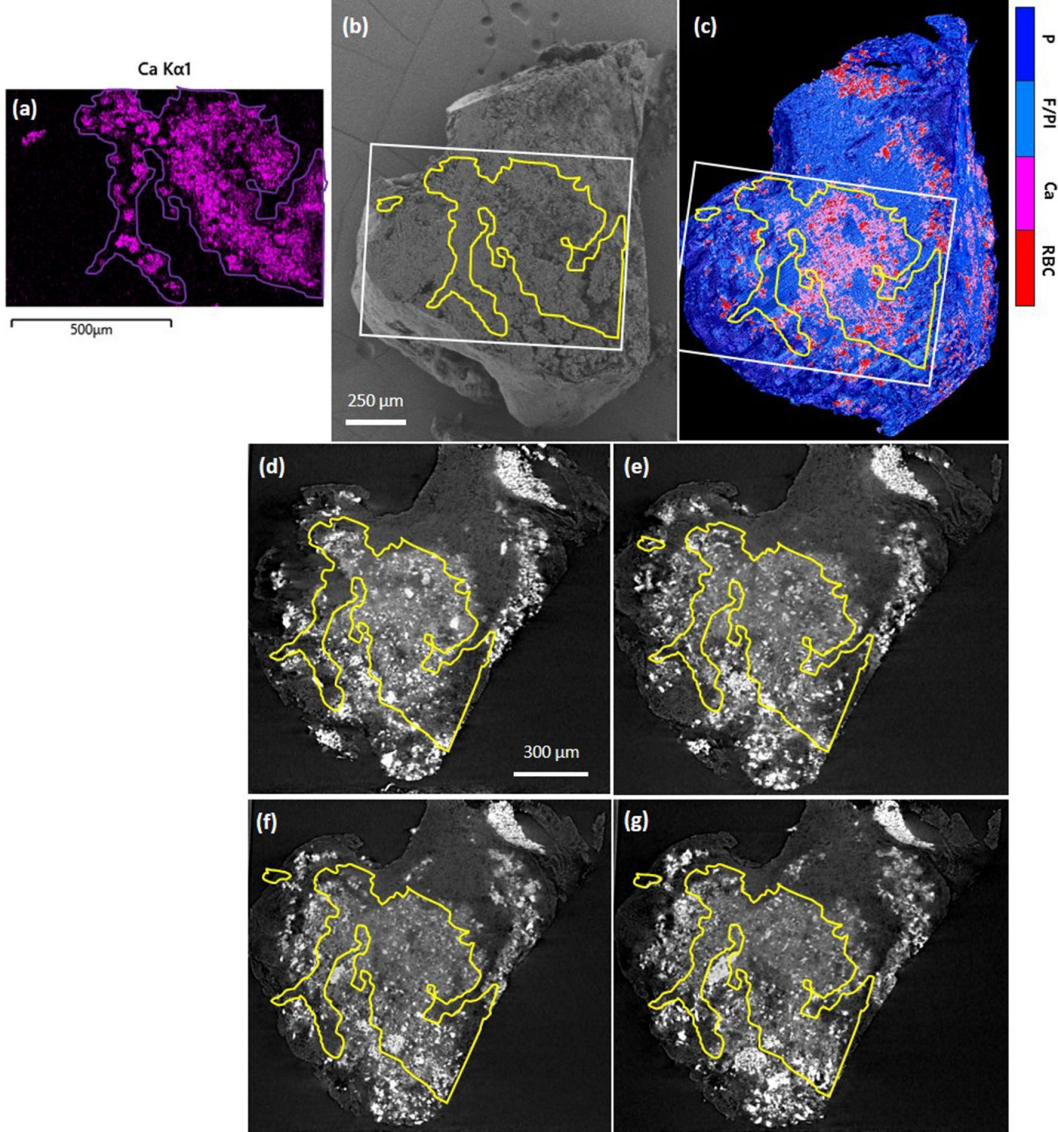
Co-registration of EDS map with microCT images for sample 3. (**a**) EDS Ca mapping segmentation. (**b**) approximate registration of EDS Ca map on SEM of the clot surface, (**c**) estimated EDS map over the segmented microCT volume, (**d**–**g**) co-registration of EDS with microCT slices corresponding to various depths within the sample from the SEM plane of view (10, 20, 30, 40 µm, respectively). White rectangle is the EDS FOV. Colorbar: P, porosity; F/Pl, fibrin/platelets; Ca, calcifications; RBC, red blood cells. In Avizo (2019.4): https://www.thermofisher.com/amira-avizo.

We performed a co-registration of EDS (and corresponding 2D SEM) with 3D rendered segmented microCT volume, as shown in Fig. 9a–c, where the 3D rendering view was adjusted to the available SEM view as close as possible. The sensitive depth of EDS measurements of this particular type of heterogeneous, porous biological sample is not feasible to be determined precisely. However, since it is expected to be large compared to the microCT voxel size, we co-registered the EDS spectra with multiple microCT slices at different depths from the plane of SEM view, as depicted in Fig. 9d–g. MicroCT slices on a plane placed on the same clot surface selected for the EDS measurement (Fig. 9b), were clipped and reconstructed down to 40 µm and then were compared to the EDS map (see Fig. 9d–g).

It should be noted that the surface of the clot was gently shaved to level it for the EDS measurement. Thus, there were certainly some materials and structures in microCT images which were removed before SEM and EDS. For this reason, we considered further depths in the new reconstruction parallel to the given clot surface to account for this difference. However, as can be observed in Fig. 9d-g, the glassy-opacity area segmented according to the gray levels conforms to the EDS map for Ca and P in various depths. This also agrees with the crystallite size of hydroxyapatite (17 nm) obtained by XRD, which is below the spatial resolution of microCT system since the calcification on microCT images is not shown as resolvable structure but appears as a glassy opacity area. However, due to the higher attenuation of X-rays for calcium than for fibrous soft tissue, although sparse, we can see more intense signal spots indicating calcifications on the microCT images. Nonetheless, this elevated gray intensity of pure nano-calcifications is still well below those related to compact RBC masses (interlaced with nano-calcifications), presumably due the iron content of the latter.

## Discussion and conclusion

According to the PB microCT results and analyses and their correlation with high-resolution SEM observations, X-ray PB microCT can provide high resolution information about the composition of blood clot samples retrieved from stroke patients. The PB microCT was particularly sensitive in detecting RBCs in the clot volumes, appeared as hyperintense structures. Even minute amounts of red blood cells can be detected, when scattered and encased in platelets or fibrins. Small and large aggregates of polyhedrocites can be reliably detected for clots in which the red blood cells are not encased in highly compact matrix (with compactness comparable or higher than the intracellular content of red blood cells). RBC shapes and sizes are consistent with the SEM observations. Fibrin dominated volumes appear at consistently low normalized CT values.

The PB microCT technique can detect the presence of polycrystalline hydroxyapatite calcifications with a glassy opacity appearance, as also proved by XRD analysis, and the mean crystallite size was quantified as a few tens of nanometers. Such calcifications on PB microCT images are detected at intensity levels between the fibrin-platelet dominated and hyperintense RBC dominated intensities, and hence, not disturbing the clot composition differentiation and analysis. However, the glassy areas are interlaced with RBCs, fibrillary strands, and platelet contents. Therefore, the presence of calcification in a clot can lead to increased average CT intensity value of all components in the biological matrix. This together with compactness variations might make it difficult to distinguish between compact and non-compact fibrin-platelet-protein regions, while yet preserving the contrast between main components, i.e. RBCs and fibrin-rich masses.

Our investigation also suggests, especially regarding the case 3, that the clinical classification of clots into three ordinal categories (red, white, mixed) based on outer optical appearance might be in certain cases misleading. Porosity volume of the clot can be calculated by PB microCT down to pore sizes of ca. two voxel units. Other potential interesting parameters e.g. fibrillary orientation and porosity distribution, and their correlations with mechanical properties of clots can be investigated in future studies on larger number of samples. Moreover, a systematic calibration of microCT systems for pure samples of RBCs, white blood cells (WBCs), and fibrins is of interest and can increase the accuracy and speed of clot analysis in future works.

Further development of automated post-processing techniques, also based on machine learning algorithms, for X-ray PB micro/nanoCT can enable high throughput analysis of blood clot composition and their 3D histological features on large sample cohorts. This can provide statistically significant information about the biological and mechanical properties of clot types, and then, implications on the relation between e.g. clot composition and MTB outcome. This is beyond reach for state-of-the art clinical 2D histology methods. Furthermore, comparison and correlation of ex-vivo PB microCT with clinical imaging methods (CT, MRI) and application of data science approaches can enable more efficient data mining and processing algorithms for the clinical imaging^12^. This can result in more precise outcome predictions that help neuro-interventionalists to make more personalized therapeutic decisions.

## Methods

### Mechanical thrombectomy

The blood clot samples analyzed in this study were collected according to the standard medical practice through MTB from patients with AIS in Neuroradiology Division of Geneva University Hospitals (HUG). The study received the ethical approval (Ref No. 2018-00476) from the Service du pharmacien cantonal, Commission cantonale d'éthique de la recherche (CCER) de Genève, for reusing the human blood clot samples and health-related data from patient victims of acute ischemic stroke. The institutional review board (CCER) waived the need for the informed consent since the effort to obtain consent from the patients was disproportionately difficult, there was no documented refusal from their part, and the research interests outweighed the interests of the person concerned (Law 34 HRA). Since our goal is to explore the capabilities of the PB microCT technique in the analysis of clot composition and structure, we selected three clots from the ones retrieved in HUG, representing each of three ordinal categories based on visual clinical expert judgement: red for the sample in Case 1, mixed appearance for the sample in Case 2, or white for the sample in Case 3. All methods were carried out in accordance with relevant guidelines and regulations.

### Sample preparation

Upon retrieval, thrombi were fixed firstly in formalin (4%) and then in glutaraldehyde (2.5%) overnight at 4 °C. Subsequently, samples were washed in a phosphate buffer solution (PBS) 10× three times for 20 min each. They were dehydrated in solutions of ascending concentrations of ethanol (50, 60, 70, 80, 90, and 100%) for 15 min each time, and were dried using critical point drying. Dried samples were kept in a desiccator containing silica gels to avoid any wet environment and structural change. The prepared samples were roughly 2 mm in width and 2.4 mm in height.

### X-ray PB micro/nano tomography

A laboratory micro/nano CT machine (RX Solutions, Chavanod, France) was utilized to acquire 3D images from each clot sample. An X-ray phase-contrast configuration was set up to also benefit from the higher contrast achievable through a propagation-based imaging. The source was a Hamamatsu L10711 micro-focus X-ray tube operated at 75 kVp and 190 µA tube current. The source spot size at this voltage peak was about 1 µm and the beam solid angle was 140°. A high-resolution CCD camera coupled to a 20-µm thick Gadox scintillator through optical fibers, formed a detection area of 36 mm × 24 mm with a pixel size of 18 µm. The detector was positioned about 24 cm from the source.

Dried clots were placed inside a Kapton tube and were padded with soft paper pieces inside the tube to restrain the electrostatic forces and avoid any motion artifact. The geometry was adjusted according to each clot size to reach the maximum possible spatial resolution. The samples were placed about 1.3–1.7 cm from the source so that typical effective voxel size varied within 1–1.5 µm for each sample. The exposure time for a single frame was 2.5 s with two averaged frames per projection. A number of 1550 projections were acquired for microCT which resulted in a total scan time of about 2.1 h. For a nanoCT analysis, samples were brought closer up to 5 mm from the source. Then, local scans were performed with higher resolution and voxel sizes down to typically 0.5 µm from designed ROIs on the clots. Propagation-based imaging was used to improve the contrast-to-noise ratio of the images based on the Paganin phase-retrieval method^13^. The phase retrieval parameters were lumped and expressed in terms of − 6 dB point of the Paganin filter which was set to 0.16 of the Nyquist frequency. This filter has been shown to be working well for the given micro/nanoCT setup and imaging parameters for soft, low-density materials^14^.

### Scanning electron microscopy

After observations with micro/nanoCT, the samples were mounted on SEM stubs using carbon tape and carbon paint and sputtered with a 5 nm AuPd (80%20%) coating. The microscopy observations were performed with an ultra-high resolution field-emission Zeiss Merlin SEM, equipped with a Gemini II column, using the Everhart–Thornley secondary electron detector, 5 keV acceleration voltage, and 500 pA probe current. EDS measurements were performed at 15 keV acceleration voltage, and the X-rays emission was recorded with an X-max80 detector.

### X-ray diffraction

X-ray diffraction patterns were measured on a Malvern Panalytical Empyrean instrument using a Debye Scherrer setup. Data collection was performed at room temperature using Ag-Kα radiation (λ = 0.5609 Å) in a range from 3 to 140° in 2Theta. The clot samples have been measured in capillaries with 1-mm diameter. Data analysis was performed using the program Highscore^15,16^. For the crystallite size determination, a Malvern Panalytical X'Pert MPD instrument with Cu-Kα (λ = 1.5406 Å) was used. The Williamson-Hall approach^17^ was used to derive the approximate hydroxyapatite crystallite sizes. Polycrystalline silicon served as the reference material for describing the instrumental peak broadening.

### Image analysis

MicroCT projections were processed and reconstructed to produce a volume dataset for each clot through XAct software (revision 21.04). It should be noted that X-ray microCT of all clot samples were performed with practically the same experimental parameters and also reconstructed using the same processing parameters and filters. To enable further inter-comparison between samples and a more generic scaling of CT gray values, we calibrated the microCT volume data in a similar way as Hounsfield Units (HU) are attributed to clinical CT pixel values. However, here we selected the air and Polystyrene (PS) as consistent reference materials (as Kapton tubes made of PS represent a very practical sample holder for the small and soft blood clot samples), instead of air and water. The mean gray level for air and Kapton tube in all reconstructed datasets were scaled to deliberate values of 500 and 1500, respectively.

Image volumes were analyzed by Avizo software (Thermo Fisher Scientific, Germany, version 2019.4), VG Studio max 3.5 (Volume Graphics), 3D Slicer (v4.11.20210226), and Matlab (R2019b) on the Image analysis platform at Empa. By manual thresholding of gray scales, confirmed by visual differentiation of structures through the volume and observed by SEM, structures inside the clot including fibrins, hyperintense RBCs, and pores were segmented for the full clot pieces. Also a 3D rendering process was performed on the segmented slices to visualize the segmented volumes. In order to verify the identified structures on the microCT, several regions of interest were selected on high resolution 2D scanning electron microcopy (SEM) images acquired in different magnifications. In Avizo, VG Studio, and 3D Slicer software, these regions were searched throughout the reconstructed microCT volume through a clipping procedure on the 3D-rendered volumes and were co-registered. Therefore, the structures were cross-checked and compared with SEM images in various views and slices. Moreover, we analyzed the segmented volumes and measured parameters such as the total volume of the clot, segmented volumes, volume fraction of each structure, and mean intensity values.

In addition, a segmentation of individual and small clusters of RBCs from the high-resolution local PB nanoCT was performed by Avizo software as well as by a method described for Purkinje cells in brain samples^18^. In the latter, an implementation of a feature-based segmentation algorithm based on the Frangi filter^19,20^ was used to identify RBCs. Originally designed for the detection of vessels, the Frangi filter provides the probability that voxel belongs to a tubular or spherical structure by analyzing the eigenvalues of the Hessian matrix. The filter was set to enhance hyperintense features as those corresponded to RBCs. Finally, the measure is the maximum value of the Frangi function within a scale range given in voxels^19^. In this case, we used a scale range of 1 to 3 voxels. Other parameters were chosen by visual inspection, and finally the noise was decreased by thresholding the values of the Frangi function at the lower 10% value.

## Supplementary Information

Supplementary Figures.
