# Author Correction: Non contrast enhanced volumetric histology of blood clots through high resolution propagation-based X-ray microtomography

**DOI:** 10.1038/s41598-022-07806-z

**Published:** 2022-03-01

**Authors:** Somayeh Saghamanesh, Daniela Dumitriu LaGrange, Philippe Reymond, Isabel Wanke, Karl‑Olof Lövblad, Antonia Neels, Robert Zboray

**Affiliations:** 1 Center for X-Ray Analytics, Swiss Federal Laboratories for Materials Science and Technology (Empa), 8600 Dübendorf, Switzerland; 2 Neuroradiology Division, Diagnostic Department, University of Geneva, Geneva, Switzerland; 3 Neuroradiology Division, Klinik Hirslanden, Zurich, Switzerland; 4 Faculty of Medicine, University of Geneva, Geneva, Switzerland

Correction to: *Scientific Reports* https://doi.org/10.1038/s41598-022-06623-8, published online 17 February 2022

The original version of this Article contained an error in the spelling of the author Daniela Dumitriu LaGrange, which was incorrectly given as Daniela Dimitriu LaGrange.

This error has now been corrected in the original Article and its accompanying Supplementary Figures file.

