# Supplementary material for: Non contrast enhanced volumetric histology of blood clots through high resolution propagation-based X-ray microtomography
Source: Sci Rep. 2022 Feb 17;12:2778. doi: 10.1038/s41598-022-06623-8 (PMC8854637; doi:10.1038/s41598-022-06623-8)
Supplement: Supplementary file 1 — Supplementary Figures. [file 41598_2022_6623_MOESM1_ESM.pdf]

Supplementary Information for

Non contrast enhanced volumetric histology of blood clots through high resolution propagation-based X-ray microtomography

Somayeh Saghamanesh [1,*], Daniela Dumitriu LaGrange [2], Philippe Reymond [2], Isabel Wanke [3], Karl-Olof Lövblad [2, 4], Antonia Neels [1], Robert Zboray [1]

[1] Center for X-ray Analytics, Swiss Federal Laboratories for Materials Science and Technology (Empa), 8600, Dübendorf, Switzerland
[2] Neuroradiology Division, Diagnostic Department, University of Geneva, Geneva, Switzerland
[3] Neuroradiology Division, Klinik Hirslanden, Zürich, Switzerland
[4] Faculty of Medicine, University of Geneva, Geneva, Switzerland
[*]somayeh.saghamanesh@empa.ch

As the two main imaging techniques used in this paper, PB microCT and SEM, cover typically quite different fields of views and resolution ranges, it is not trivial to find direct correspondence between them. This is especially the case for biomedical samples involving complicated irregular shapes and soft structures that can be slightly obstructed by e.g. sample transport and positioning. We further demonstrate here direct correspondence of the clots structures that can be identified in specific ROIs using micro CT PBI over different scales as well as their corresponding intensity values with those that are obtained via cascades of SEM images with different resolution and field of views. To this end, visual inspection of structural landmarks have been used to identify corresponding areas/volumes. In addition to Fig. 3 in the paper, this is shown in Fig. S1 and Fig. S2 for sample 1 and sample 3, respectively.

The segmentation of RBCs and overall structures in Fig. S1 was based on the hyper-intense gray value range confirming that RBCs show up as small hyper-intense volumes in the microCT PBI images. The threshold for the isosurface rendering was placed at around the mid value of the intensity range describing loose fibrin-dominated region to enable visualization of overall clot surface structures and landmarks. As it is the lowest intensity of all structures, it includes and shows all the structures (RBCs, compact fibrin etc.) on the clot surface. This isosurface is shown in partly transparent gray in the Fig. S1-b and S1-d as a tradeoff to visualize both the three selected RBCs and the landmark features. Similarly, the three selected RBCs shown in red in Fig. S1-d were segmented based on a threshold placed around the mid value of the intensity range established for RBCs. These are shown in the figure in non-transparent red for better visibility. Note that achieving a perfect match of the perspective of the SEM and CT surface rendering is practically impossible, also due to subtle changes of sample surface when transporting, handling, and imaging. Nonetheless, Fig. S1-c and S1-d prove that we find a very clear correspondence between microCT and SEM even for individual RBCs on the surface.

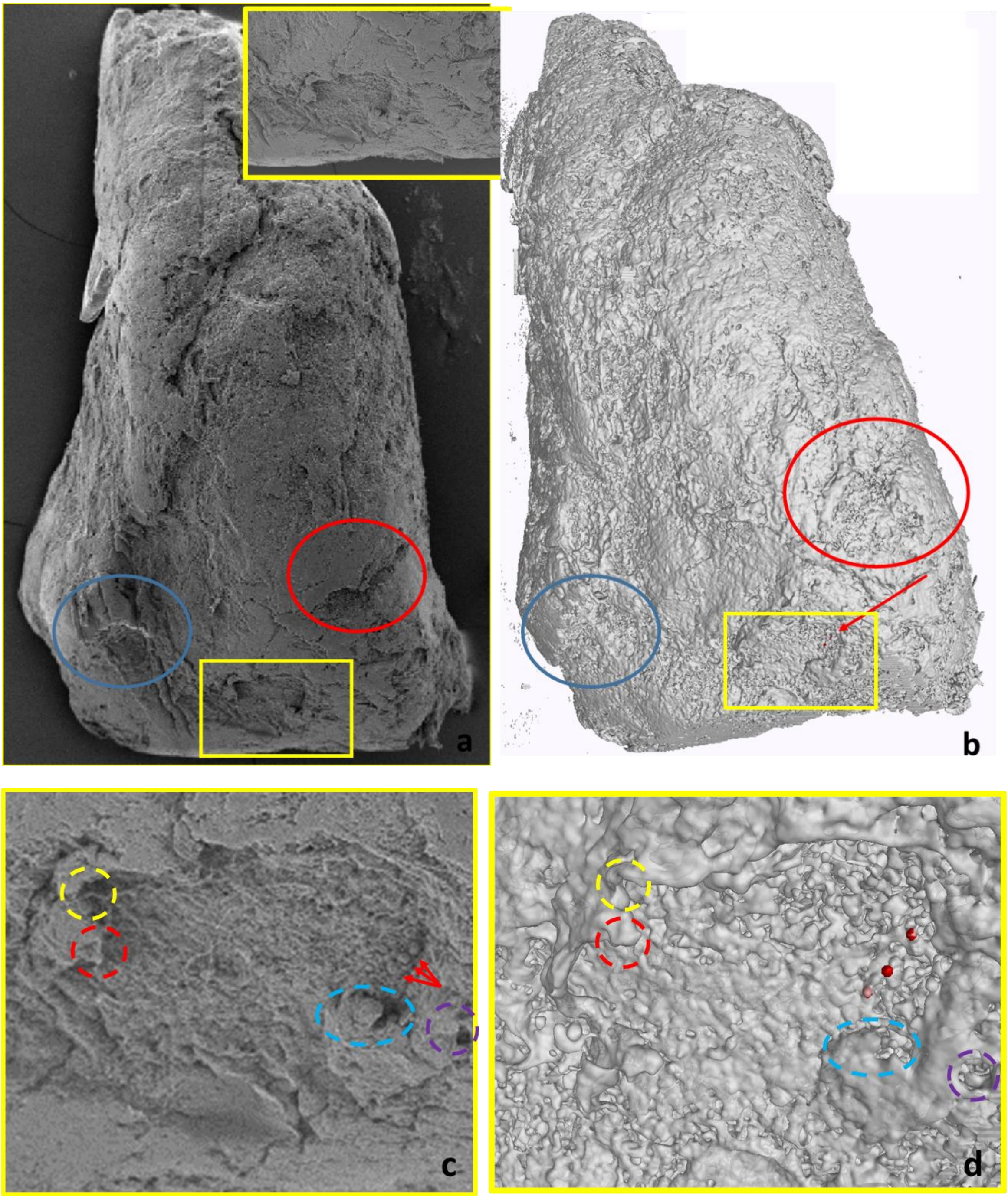

Figure S1. Correspondence between three well-separated, individual RBCs n the clot surface on the SEM and PB microCT images. (a) SEM image and (b) 3D volume rendering of the full clot surface segmented from microCT based on the average intensity level for loose fibrin region, being the lowest of all, thus containing all surface structures. The (red, blue, yellow) contours show corresponding characteristic surface structures used as landmarks for the approximate registration of the images. (c, d) show zoomed ROI denoted by the yellow rectangle in (a , b). The three red arrows indicate the three well-separated RBCs on the clot surface in (b, c, d). These are segmented out based on the average RBCs intensity level and shown in non-transparent red in the CT 3D rendering. In VGStudio max (3.5.0): https://www.volumegraphics.com.

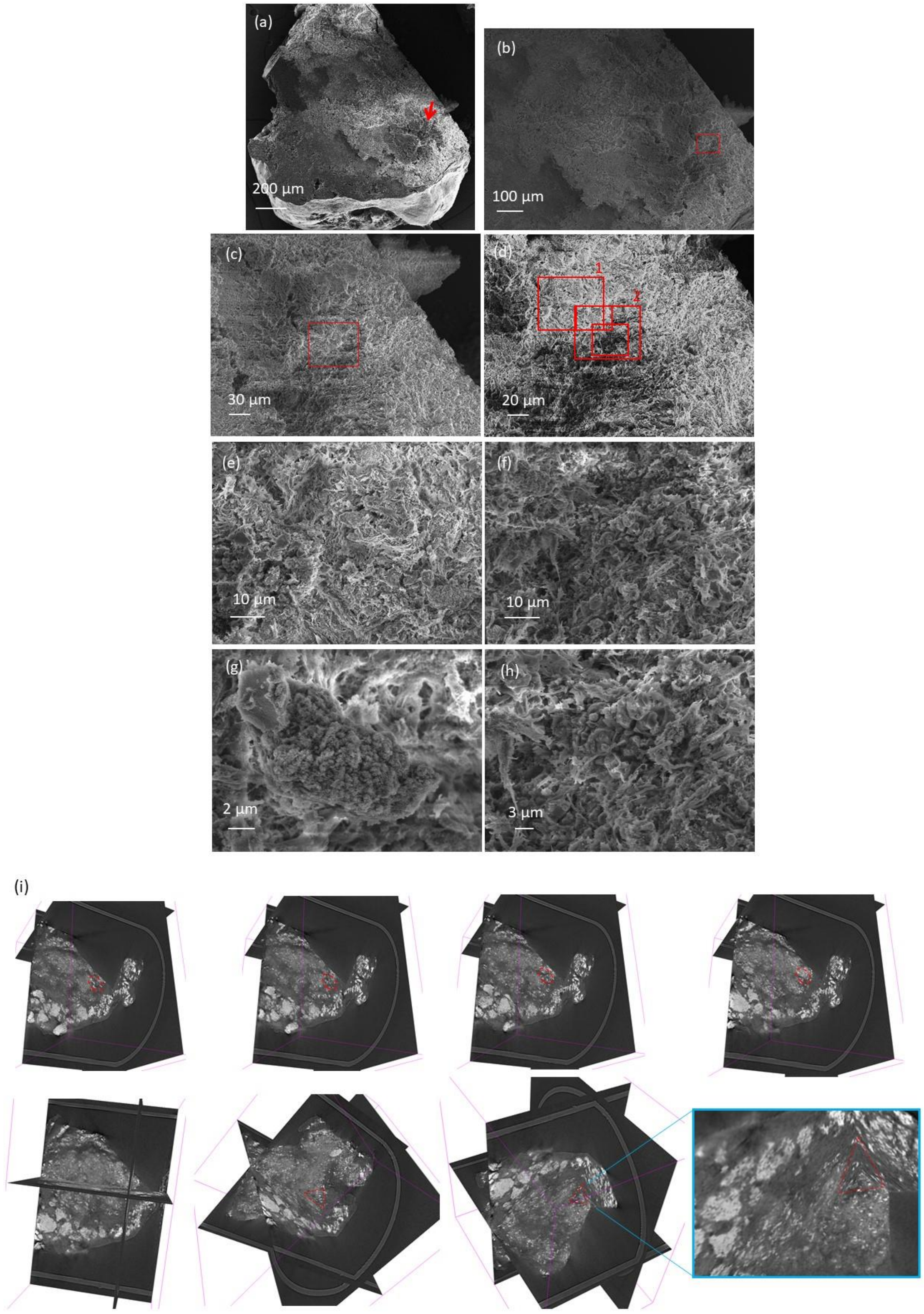

Figure S2. (a-h) illustration of localization of regions of interest in SEM micrographs of various magnifications. (e) corresponds to area marked with red rectangle 1 in (d), (f) corresponds to area marked with red rectangle 2 in (d), and (g, h) are the insets in red rectangle 2. (i) localization of the region of interest using 3D Slicer software (v. 4.11.20210226): https://www.slicer.org/. SEM images were generated by ZeissSmartSEM software (version 6.06).

For attributing the CT findings to biological structures existent within the bulk of the sample we adopted the following approach.

1- The surface of various cuts of the clot was inspected with SEM, as such that we can access the bulk of the clot for which we have 3D information from CT images, and provide information on various biological structures and organization.

2- Certain fiducial landmarks were determined on the clot sample, such as distinct notches, peaks, angles, surface shape, etc, recognizable on both SEM and 3D CT slices (e.g. see yellow arrows in Fig. 3-a,d, dashed contours in Fig. S1, or red arrow in Fig. S2-a, for such fiducial landmarks).

3- The samples were cut after the CT data were recorded. Then, the surface of the cuts were sampled with SEM.

4- Using the landmarks naturally occurring in the sample and identifiable with both techniques (CT and SEM), the corresponding CT volume images were searched to find those landmarks and the specific depth image were matched to the SEM image as close as possible. Next, we reconstructed the CT slice at the same view and depth as the acquired SEM images. The reconstructed microCT images were segmented through our thresholding approach and co-registered with the SEM images. The visualized structures (RBCs, fibrin/platelet, porosity) on SEM were consistent with relevant segmentations (colors) on co-registered CT-SEM images.

In Fig. S2 is illustrated the process of spatial correlation, which involves scrolling within the 3D volume of the CT scan in orthogonal directions, and localization of the point of observation in SEM within a region of the CT scan. The scales in SEM and CT images were used to estimate the distances using 3DSlicer software (v4.11), and the pits at the border of the sample served as landmarks. Low-magnification images were used to perform the spatial correlation (placement within a CT macro-domain), high resolution SEM images were used to identify the biological structures and organization within macro-domains.
